# Supplementary material for: Correction of a homoplasmic mitochondrial tRNA mutation in patient-derived iPSCs via a mitochondrial base editor
Source: Commun Biol. 2023 Nov 3;6:1116. doi: 10.1038/s42003-023-05500-y (PMC10624837; doi:10.1038/s42003-023-05500-y)
Supplement: Supplementary file 2 — Supplementary Information [file 42003_2023_5500_MOESM2_ESM.pdf]

Supplementary information

Correction of a homoplasmic mitochondrial tRNA mutation in patient-derived iPSCs via a mitochondrial base editor

Xiaoxu Chen[1#], Mingyue Chen[1#], Yuqing Zhu[2#], Haifeng Sun[1#], Yue Wang[3#], Yuan Xie[4], Lianfu Ji[5], Cheng Wang[4], Zhibin Hu[6], Xuejiang Guo[3], Zhengfeng Xu[2]*, Jun Zhang[1]*, Shiwei Yang[5]*, Dong Liang[2]*, Bin Shen[1, 6]*

Contents

Supplementary Figure 1. m.A4300G mutation causes maternally inherited hypertrophic cardiomyopathy.

Supplementary Figure 2. Characteristics of m.A4300G-iPSCs and its derived cardiomyocytes.

Supplementary Figure 3. DdCBE-mediated editing in HEK293FT and iPSCs.

Supplementary Figure 4. Analysis of genetically corrected iPSCs.

Supplementary Figure 5. DdCBE-mediated off-target editing in whole mtDNA of iPSC clones with 0-5%, 5-30%, 30-60% and over 60% editing efficiencies.

Supplementary Figure 6. Off-target analysis in mitochondrial and nuclear DNA and mt-gene expression analysis.

Supplementary Figure 7. PRM analysis of mt-gene expression.

Supplementary Figure 8. Gating strategy for EGFP/mCherry double positive cells.

Supplementary Figure 9. Uncropped western blot images.

Supplementary Table 1. List of excluded SNPs.

Supplementary Table 2. Isotope-labeled heavy synthetic peptide sequence used for relative targeted quantification by PRM.

Supplementary Table 3. Primer Information.

Supplementary Note

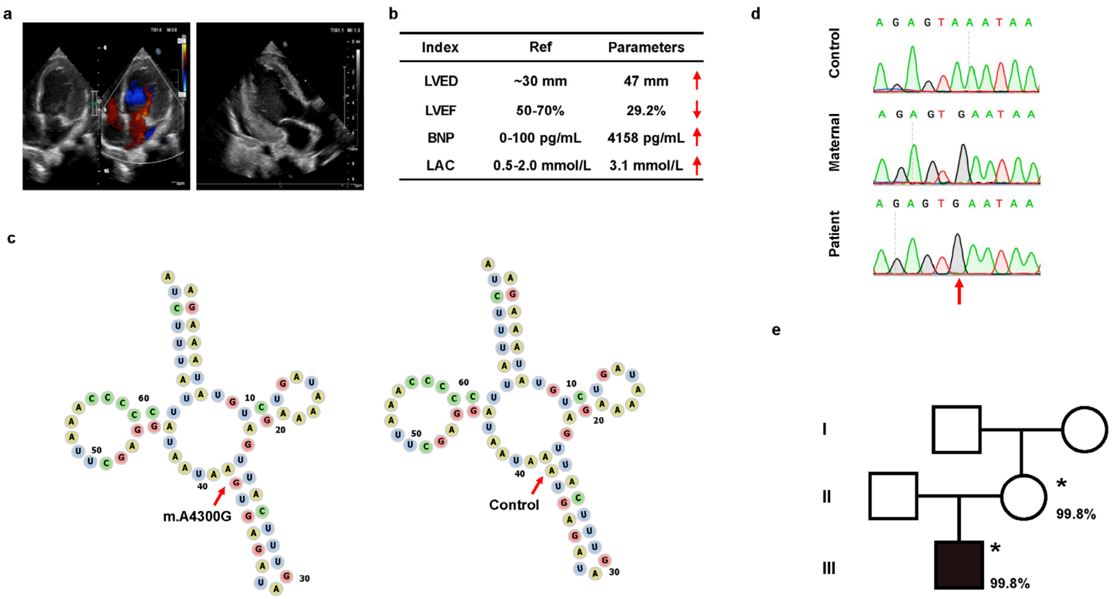

Supplementary Figure 1. m.A4300G mutation causes maternally inherited hypertrophic cardiomyopathy.

a. Echocardiography revealed enlarged left ventricle (left) and slightly thickened septum and posterior left ventricular wall (right).

b. Clinical characteristics of the patient. LVED: left ventricular end-diastolic diameter, LVEF: left ventricular systolic function, BNP: B-type natriuretic peptide, LAC: lactic acid.

c. Illustration of mt-tRNA^{Ile} bearing the m.A4300G mutation and a wild type control.

d. Sequence chromatograms around m.A4300 site in PBMC of control, maternal and patient. Red arrow indicates mutant site.

e. Pedigrees of patient. Solid symbol indicates clinically affected individual, and those tested for m.4300A>G mutation by deep sequencing have an asterisk.

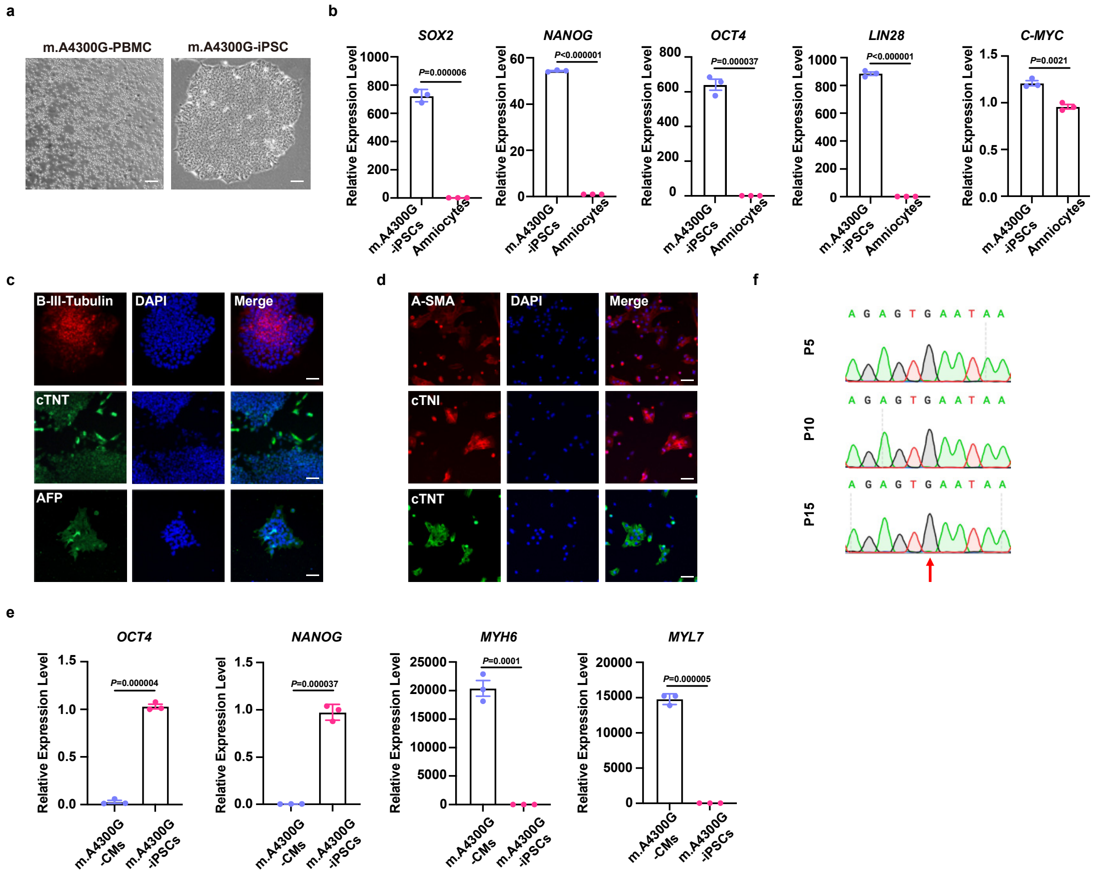

Supplementary Figure 2. Characteristics of m.A4300G-iPSCs and its derived cardiomyocytes.

a. The phase images of PBMCs and a m.A4300G-iPSC clone from a patient with m.A4300G mutation. Scale bars, 50 μm.

b. qRT-PCR verification of pluripotency markers *SOX2* (Effect size = 721.88), *NANOG* (Effect size = -53.34), *OCT4* (Effect size = -638.47), *LIN28* (Effect size = 883.87) and *C-MYC* (Effect size = -0.25) in m.A4300G-iPSCs and amniocytes. Data are presented as mean ± SEM, n = 3 independent experiments.

c. Immunofluorescence staining of ectoderm marker (B-III-Tubulin), mesoderm marker (cTNT) and endoderm marker (AFP) in the three germ layers derived from m.A4300G-iPSCs. Scale bars, 50 μm.

d. Immunofluorescence staining of cardiomyocyte markers A-SMA, cTNI and cTNT in cardiomyocytes derived from m.A4300G-iPSCs. Scale bars, 50 μm.

e. qRT-PCR verification of *OCT4* (Effect size = 1.0001), *NANOG* (Effect size = 0.9689), *MYH6* (Effect size = -20409) and *MYL7* (Effect size = -14790) expression

in m.A4300G-iPSCs and m.A4300G-iPSC derived cardiomyocytes (m.A4300G-CMs). Data are presented as mean ± SEM; n = 3 independent experiments.

f. Sequence chromatograms of m.A4300G mutation at different passages. Red arrow indicates mutant site.

Significance was calculated with unpaired two-tailed Student's *t*-test.

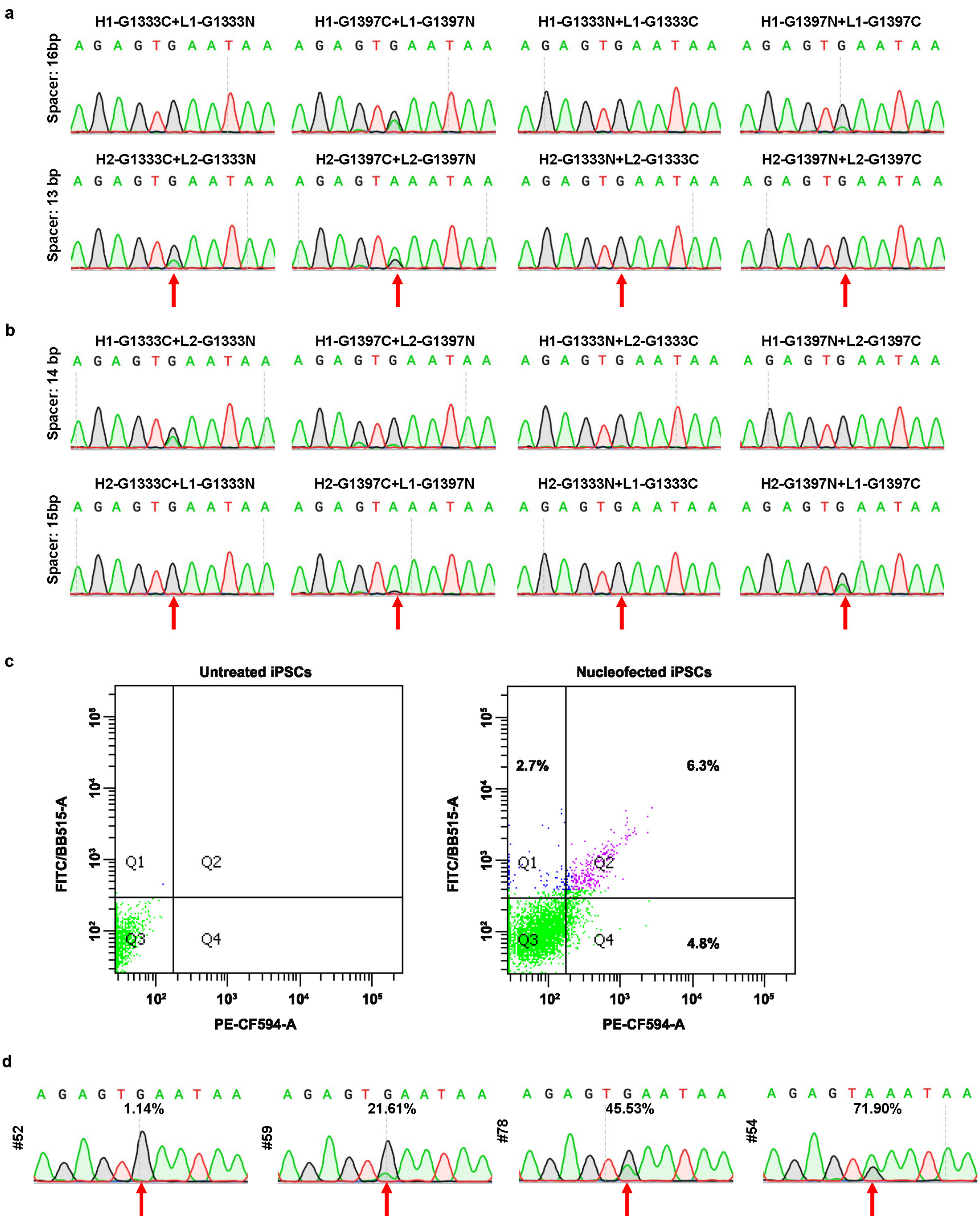

Supplementary Figure 3. DdCBE-mediated editing in HEK293FT and iPSCs.

a. Sanger sequencing results of the first round screening for H1-DdCBE + L1-DdCBE pair with 16 bp spacing region and H2-DdCBE+L2-DdCBE with 13 bp spacing region.

b. Sanger sequencing results of the second round screening of H1-DdCBE + L2-DdCBE pair with 14 bp spacing region and H2-DdCBE+L1-DdCBE pair with 15 bp spacing region.

c. Flow cytometry of untreated iPSCs and nucleofected iPSCs. Purple dots indicate the sorted cells for culture.
d. Representative sequence chromatograms of clones in 0-5%, 5-30%, 30-60% and over 60% groups.

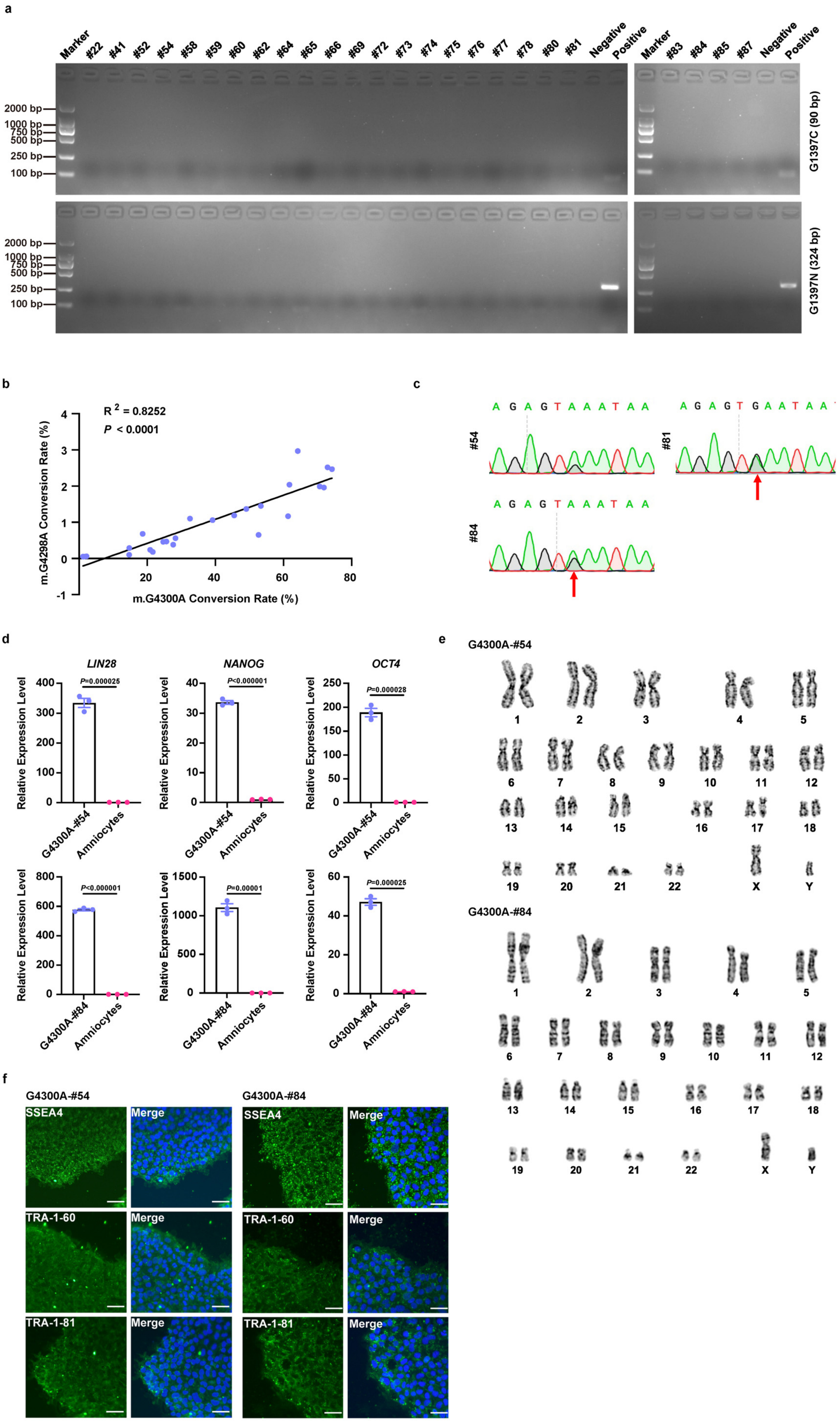
a
Marker #22 #41 #52 #54 #58 #59 #60 #62 #64 #65 #66 #69 #72 #73 #74 #75 #76 #77 #78 #80 #81 Negative Positive
Marker #83 #84 #85 #87 Negative Positive
2000 bp
1000 bp
750 bp
500 bp
250 bp
100 bp
G1397C (90 bp)
G1397N (324 bp)
b
R 2 = 0.8252
P < 0.0001
m.G4298A Conversion Rate (%)
m.G4300A Conversion Rate (%)
c
#54
#81
#84
A G A G T A A A T A A
A G A G T G A A T A A
d
LIN28
NANOG
OCT4
Relative Expression Level
P=0.000025
P<0.000001
P=0.000028
P<0.000001
P=0.00001
P=0.000025
G4300A-#54
G4300A-#84
Amniocytes
e
G4300A-#54
G4300A-#84
X
Y
f
G4300A-#54
G4300A-#84
SSEA4
TRA-1-60
TRA-1-81
Merge

Supplementary Figure 4. Analysis of genetically corrected iPSCs.

a. Detection of the integrated $DddA_{tox}$ halves in the genome of genetically corrected iPSC clones.

b. Correlations between on-target editing efficiencies and bystander editing efficiencies in edited clones.

c. Sanger sequencing results of #54, #81 and #84 clones at passage 20.

d. qRT-PCR verification of pluripotency markers *LIN28* (Effect size = 333.3), *NANOG* (Effect size = -32.62) and *OCT4* (Effect size = -188.1) in G4300A-#54 vs. amniocytes; *LIN28* (Effect size = -575.6), *NANOG* (Effect size = -46.10) and *OCT4* (Effect size = -1103) in G4300A-#84 vs. amniocytes. Data are presented as mean ± SEM; n = 3 independent experiments.

e. The karyotype of G4300A-#54 and #84.

f. Immunofluorescence staining of pluripotency markers SSEA4, TRA-1-60 and TRA-1-81 in G4300A-#54 and #84. Scale bars, 50 μm.

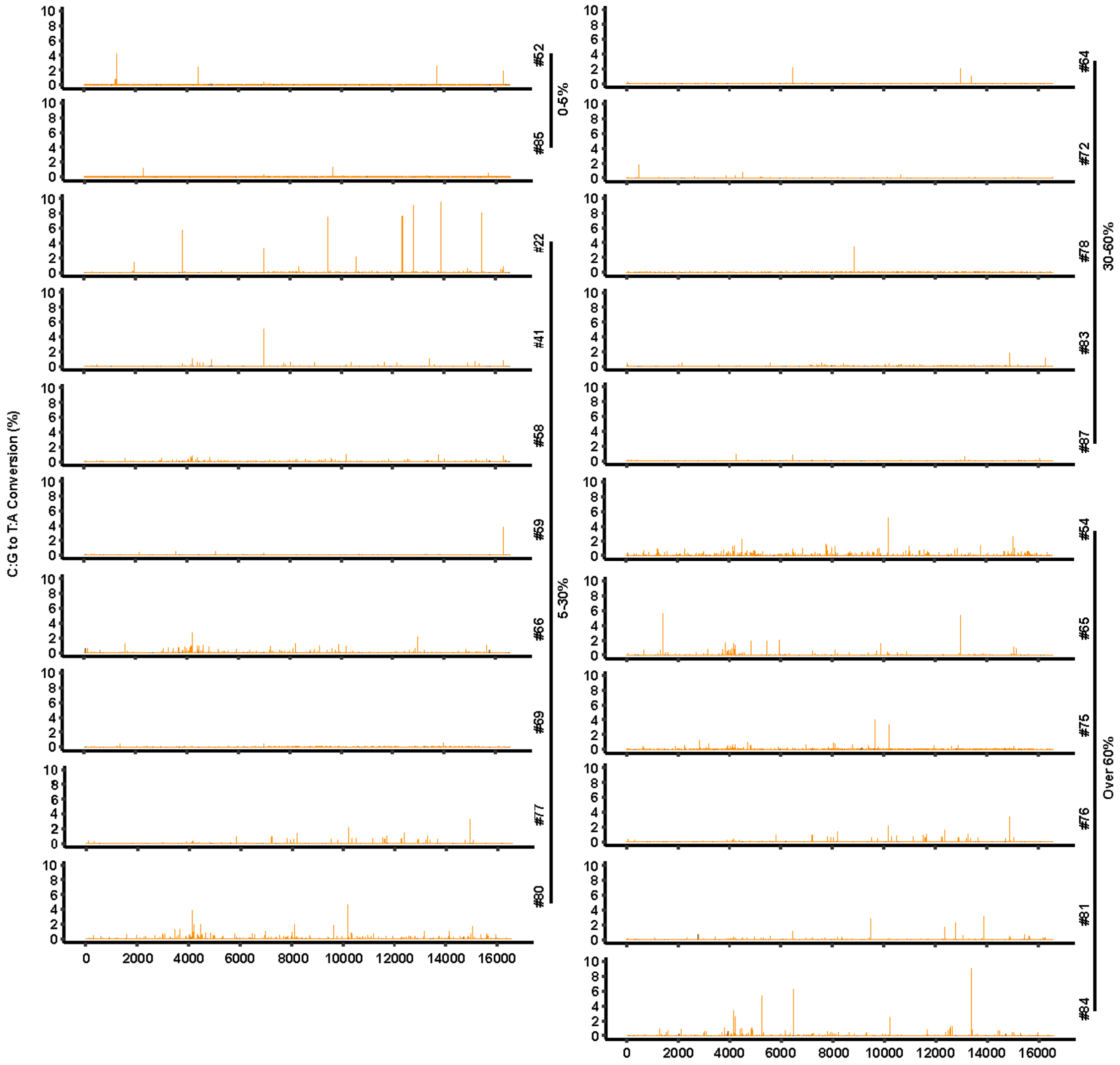

Supplementary Figure 5. DdCBE-mediated off-target editing in whole mtDNA of iPSC clones with 0-5%, 5-30%, 30-60% and over 60% editing efficiencies.

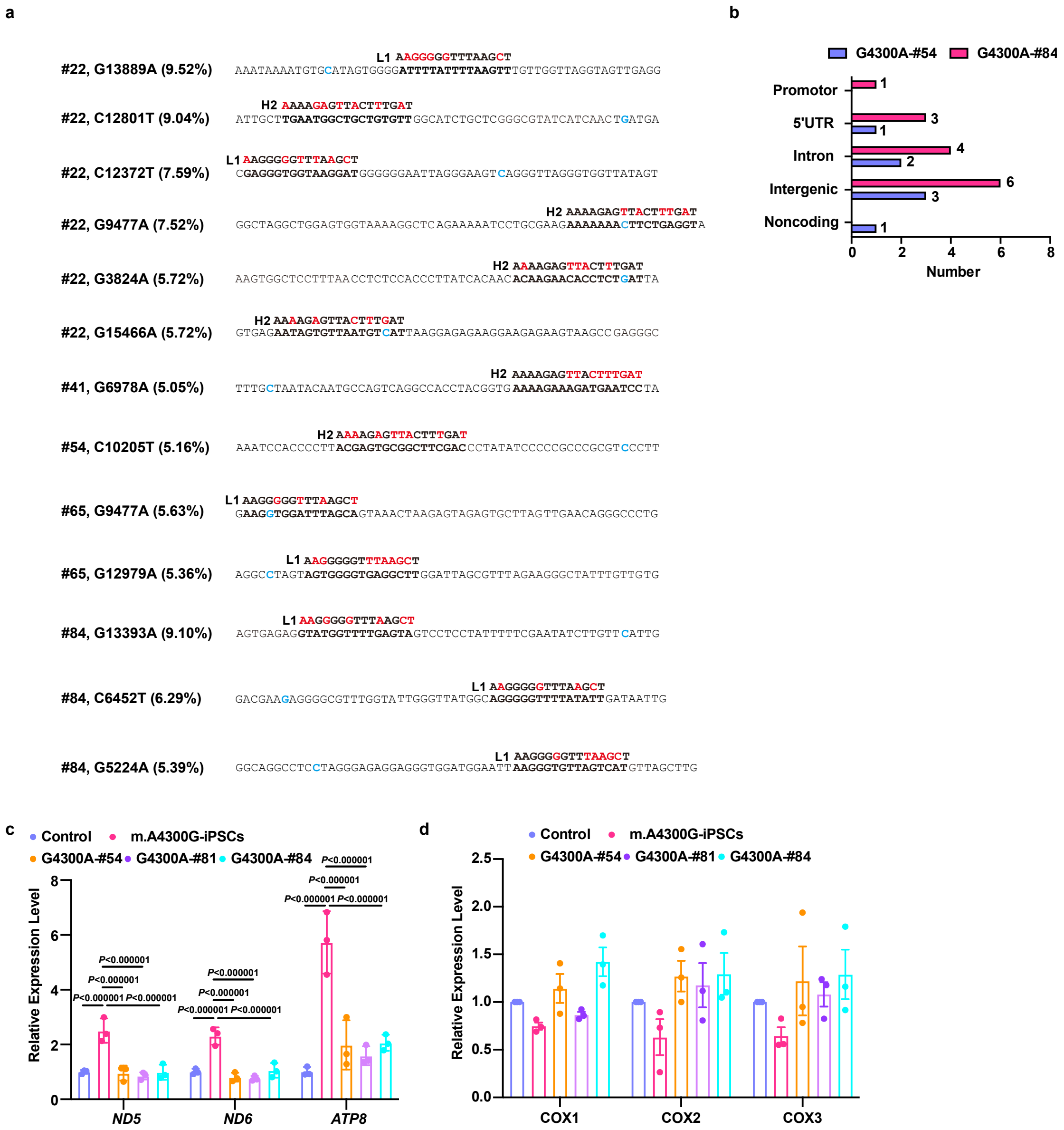

Supplementary Figure 6. Off-target analysis in mitochondrial and nuclear DNA and mt-gene expression analysis.

a. Analysis of off-target sites with editing efficiencies over 5% induced by DdCBE in a sequence dependent or independent manner. Unmatched bases are marked in red. G-A pairs also be marked in red because G-RVD recognizes A base simultaneously. Off-target site is in blue.

b. Location of C·G-to-T·A SNVs in nuclear DNA of #54 and #84 clones.

c. qRT-PCR verification of *ND5, ND6* and *ATP8* expression level in control, m.A4300G-iPSCs and corrected iPSC clones. Effect size for Control vs. m.A4300G-iPSCs, m.A4300G-iPSCs vs. G4300A-#54, m.A4300G-iPSCs vs.

G4300A-#81 and m.A4300G-iPSCs vs. G4300A-#84 are -0.5933, 1.163, 1.287 and 1.093 in *ND5*; -0.7500, 1.290, 1.417 and 1.180 in *ND6*; -0.5267, 1.103, 1.186 and 0.977 in *ATP8*. Data are presented as mean ± SEM, n = 3 independent experiments. Significance was calculated with One-way ANOVA.

d. The quantitative analysis of *COX1*, *COX2* and *COX3* protein level in control, m.A4300G-iPSCs and corrected iPSC clones. Data are presented as mean ± SEM， n = 3 independent experiments.

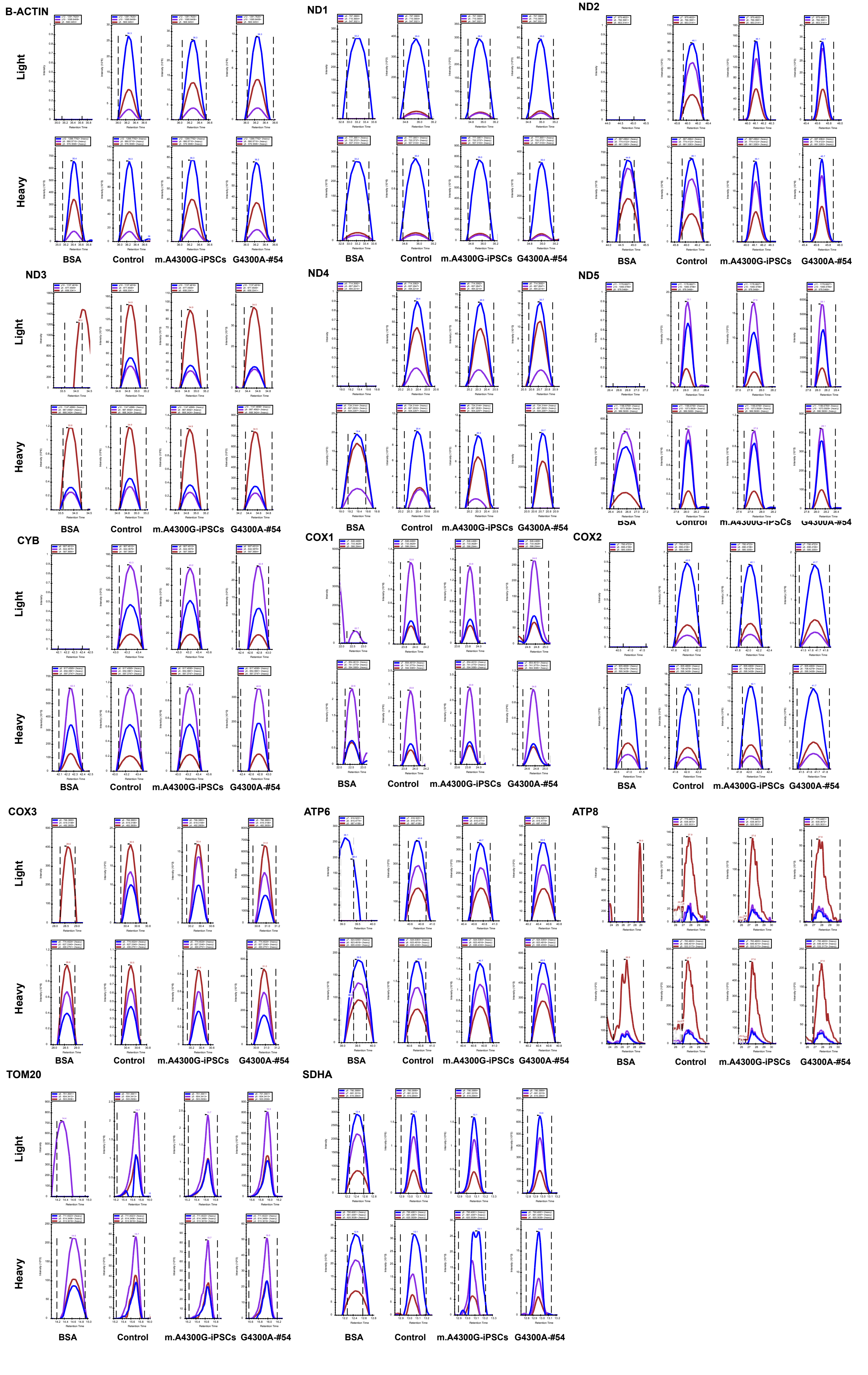

B-ACTIN
ND1
ND2
ND3
ND4
ND5
CYB
COX1
COX2
COX3
ATP6
ATP8
TOM20
SDHA
Light
Heavy
BSA
Control
m.A4300G-iPSCs
G4300A-#54

Supplementary Figure 7. PRM analysis of mt-gene expression.

Representative images of extracted ion chromatograms by relative protein quantification using PRM. Light and heavy individually indicated endogenous protein counterparts and its corresponding isotope-labeled synthetic peptides with ^{13}C and ^{15}N on C-terminal arginine or lysine. The tryptic peptides of bovine serum albumin (BSA) spiked with isotope-labeled heavy peptides served as negative control.

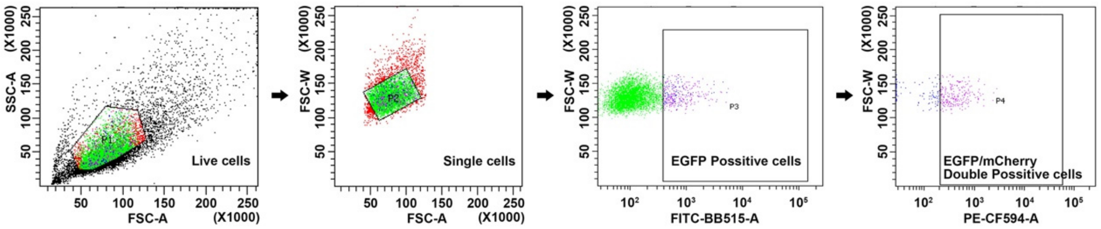

Supplementary Figure 8. Gating strategy for EGFP/mCherry double positive cells.

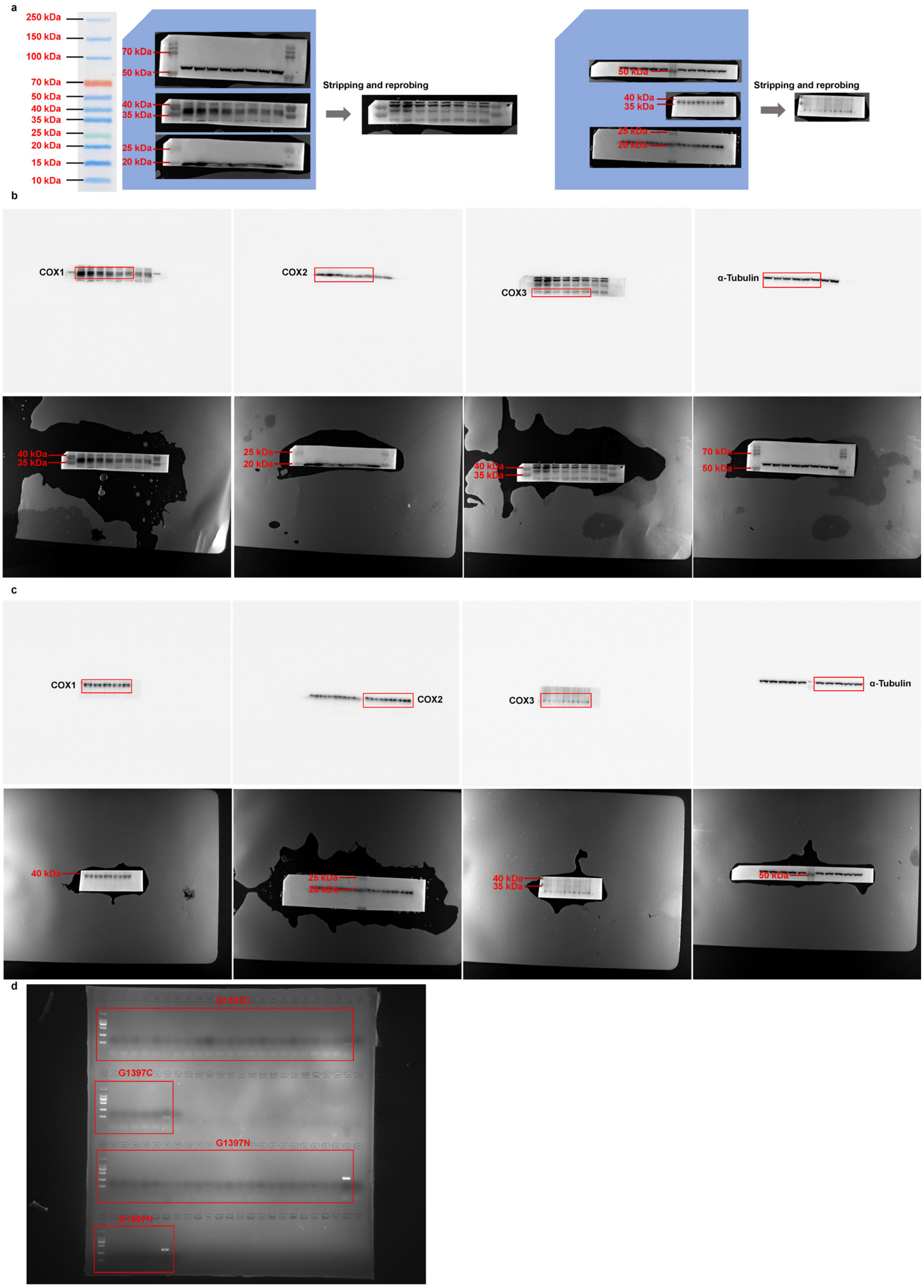

Supplementary Figure 9. Uncropped western blot images.

a. Schematic diagram shows that all vertically stacked strips of bands are derived from the same membrane. COX3 was detected by stripping and reprobing the same membrane of COX1.

b. Uncropped western blot images of Figure 1h. The bands in red boxes are presented in Figure 1h.
c. Uncropped western blot images of Figure 4e. The bands in red boxes are presented in Figure 4e.
d. Uncropped image for the gel presented in Supplementary Figure 4a. The bands in red boxes are presented in Supplementary Figure 4a.

Supplementary Table 1. List of excluded SNPs.

Excluded SNPs
chrMT_7028_C
chrMT_11719_G
chrMT_6531_C
chrMT_14766_C
chrMT_739_C
chrMT_16223_C
chrMT_15301_G
chrMT_15043_G
chrMT_12705_C
chrMT_10400_C
chrMT_11969_G
chrMT_7642_G
chrMT_14340_C
chrMT_11518_G
chrMT_858_C

Supplementary Table 2. Isotope-labeled heavy synthetic peptide sequence used for relative targeted quantification by PRM.

Gene name	Peptide sequence	Precursor ion (m/z)
ND1	ILGYMQLR ($^{13}C_6$,$^{15}N_4$)	502.2853
ND2	WAIIEEFTK ($^{13}C_6$,$^{15}N_2$)	572.8101
ND3	STPYECGFDPMSPAR ($^{13}C_6$,$^{15}N_4$)	862.8678
ND4	LGGYGMMR ($^{13}C_6$,$^{15}N_4$)	447.7136
ND5	TISQHQISTSIITSTQK ($^{13}C_6$,$^{15}N_2$)	941.012
CYB	DVNYGWIIR ($^{13}C_6$,$^{15}N_4$)	573.3025
COX1	WLFSTNHK ($^{13}C_6$,$^{15}N_2$)	520.7738
COX2	VVLPIEAPIR ($^{13}C_6$,$^{15}N_4$)	558.8544
COX3	QLMFHFTSK ($^{13}C_6$,$^{15}N_2$)	382.8667
ATP6	LITTQQWLIK ($^{13}C_6$,$^{15}N_2$)	626.3812
ATP8	MLNTNYHLPPSPK ($^{13}C_6$,$^{15}N_2$)	507.2654
TOM20	LPTISQR ($^{13}C_6$,$^{15}N_4$)	412.7468
SDHA	NTVVATGGYGR ($^{13}C_6$,$^{15}N_4$)	552.7872
ACTB	VAPEEHPVLLTEAPLNPK ($^{13}C_6$,$^{15}N_2$)	981.5429

Supplementary Table 3. Primer Information

	Primers	Sequence (5'-3')
**Primers for RVD assembly**	RVD seq Fwd	TGACCGCAGTGGAGGCAGTG
	RVD seq Rev	TTCACTGCATCCAGCGCAGG
**Sythesized Oligo**	homo-m.4300-Sense	CTAAGAAATATGTCTGATAAAAGAGTTACTTTGATA GAGTGAATAATAGGAGC
	homo-m.4300-Antisense	TTCTCATAGTCCTAGAAATAAGGGGGTTTAAGCTCC TATTATTCACTCTATCAA
**Primers for genotyping and PCR1**	homo-m.4300-Fwd	AGCAGTAGCCCAAACAATCTCA
	homo-m.4300-Rev	GCTAAGATTTTGCGTAGCTGGG
	1397C-For	GCAATCCCTGTGAAGAGAGGC
	1397C-Rev	GCAGCCGCCCTTTGTTGG
	1397N-For	GGCTCCTACGCCCTGGGCC
	1397N-Rev	TCCCTCAGGTGGCACCACTGTC
	T-A4300G-Fwd	GATCACTCTCGGCATGGACG
	T-A4300G-Rev	ACAAACCACAACTAGAATGCAGTG
**Barcoded Primers for PCR2**	i5-homo- m.4300-Fwd1	ACACTCTTTCCCTACACGACGCTCTTCCGATCTGAG AATCTCCAGCATTCCCCCTCAAA
	i5-homo- m.4300-Fwd2	ACACTCTTTCCCTACACGACGCTCTTCCGATCTTAAA ATCTCCAGCATTCCCCCTCAAA
	i5-homo- m.4300-Fwd3	ACACTCTTTCCCTACACGACGCTCTTCCGATCTTCAA ATCTCCAGCATTCCCCCTCAAA
	i5-homo- m.4300-Fwd4	ACACTCTTTCCCTACACGACGCTCTTCCGATCTTCGA ATCTCCAGCATTCCCCCTCAAA
	i5-homo- m.4300-Fwd5	ACACTCTTTCCCTACACGACGCTCTTCCGATCTTGCA ATCTCCAGCATTCCCCCTCAAA
	i7-homo-m.4300-Rev	GTGACTGGAGTTCAGACGTGTGCTCTTCCGATCTAC CGGATTCTCAGGGATGGGTTCGAT
	T-i5-Fwd1	ACACTCTTTCCCTACACGACGCTCTTCCGATCTCAAG ATCACTCTCGGCATGGACG
	T-i5-Fwd2	ACACTCTTTCCCTACACGACGCTCTTCCGATCTTCAG ATCACTCTCGGCATGGACG
	T-i5-Fwd3	ACACTCTTTCCCTACACGACGCTCTTCCGATCTACCG ATCACTCTCGGCATGGACG
	T-i5-Fwd4	ACACTCTTTCCCTACACGACGCTCTTCCGATCTGTAG ATCACTCTCGGCATGGACG
	T-i5-Fwd5	ACACTCTTTCCCTACACGACGCTCTTCCGATCTCAGG ATCACTCTCGGCATGGACG
	T-i7-Rev	GTGACTGGAGTTCAGACGTGTGCTCTTCCGATCTAC AAACCACAACTAGAATGCAGTG
	homo-mtDNA-F1-Fwd	GCAAATCTTACCCCGCCTG

**Primers for long-range PCR**	homo-mtDNA-F1-Rev	AATTAGGCTGTGGGTGGTTG
	homo-mtDNA-F2-Fwd	GCCATACTAGTCTTTGCCGC
	homo-mtDNA-F2-Rev	GGCAGGTCAATTTCACTGG
**Stem-loop Primers for tRNA reverse transcription**	homo-stem-loop-<br>m.tRNA^{Ile}-Rev	GTCGTATCCAGTGCAGGGTCCGAGGTATTCGCACTG<br>GATACGACTAGAAA
	homo-stem-loop-<br>m.tRNA^{Leu}-Rev	GTCGTATCCAGTGCAGGGTCCGAGGTATTCGCACTG<br>GATACGACTGTTAA
**Primers for qRT-PCR**	m.tRNA^{Ile}-Fwd	ATAATAGGAGCTTAAACCCCCTTA
	m.tRNA^{Ile}-Rev	AGTGCAGGGTCCGAGGTATT
	m.tRNA^{Leu}-Fwd	CAGTCAGAGGTTCAATTCCTCTTC
	m.tRNA^{Leu}-Rev	AGTGCAGGGTCCGAGGTATT
	homo-U6-Fwd	CTCGCTTCGGCAGCACA
	homo-U6-Rev	AACGCTTCACGAATTTGCGT
	homo-ND5-Fwd	GAACAAGATATTCGAAAAATAGGAGGAC
	homo-ND5-Rev	GCGGTTTCGATGATGTGG
	homo-ND6-Fwd	CACTCACCAAGACCTCAACC
	homo-ND6-Rev	GAATGATGGTTGTCTTTGGATATACTAC
	homo-ATP8-Fwd	CTTACACTATTCCTCATCACCCAAC
	homo-ATP8-Rev	GTTCATTTTGGTTCTCAGGGTTTG
	homo-COX1-Fwd	AAGCCTCCTTATTCGAGCCG
	homo-COX1-Rev	GGGGGCACCGATTATTAGGG
	homo-COX2-Fwd	GTACTCCCGATTGAAGCCCC
	homo-COX2-Rev	ACCGTAGTATACCCCCGGTC
	homo-COX3-Fwd	TCACCCCGCTAAATCCCCTA
	homo-COX3-Rev	CGTCGGAAATGGTGAAGGGA
	homo-B2M-Fwd	CAGGTACTCCAAAGATTCAGG
	homo-B2M-Rev	GTCAACTTCAATGTCGGATGG

Supplementary Note

Amino acid sequences of DdCBE used are annotated as: red for MTS, italics for linker, yellow for flag tag, green for N&C-terminal domain, underlined for RVDs, purple for split $DddA_{tox}$ halves, cyan for UGI. Only RVDs sequences are showed for H2-, L1- and L2- DdCBEs.

H1-G1333C

MLGFVGRVAAAPASGALRRLTPSASLPPAQLLLRAAPTAVHPVRDYAAQTSESGGGG
SPG*AAA*DYKDDDDK*GS*VDLRTLGYSQQQQEKIKPKVRSTVAQHHEALVGHGFTHAHI
VALSQHPAALGTVAVKYQDMIAALPEATHEAIVGVGKQWSGARALEALLTVAGELRG
PPLQLDTGQLLKIAKRGGVTAVEAVHAWRNALTGAPLNLTPEQVVAIASNIGGKQAL
ETVQRLLPVLCQAHGLTPDQVVAIASNIGGKQALETVQRLLPVLCQAHGLTPDQVVA
IASNIGGKQALETVQRLLPVLCQAHGLTPDQVVAIASNIGGKQALETVQRLLPVLCQ

AHGLTPDQVVAIASNNGGKQALETVQRLLPVLCQAHGLTPEQVVAIASNIGGKQALE
TVQRLLPVLCQAHGLTPDQVVAIASNNGGKQALETVQRLLPVLCQAHGLTPEQVVAI
ASNGGGKQALETVQRLLPVLCQAHGLTPEQVVAIASNGGGKQALETVQRLLPVLCQA
HGLTPEQVVAIASNIGGKQALETVQRLLPVLCQAHGLTPDQVVAIASHDGGKQALET
VQRLLPVLCQAHGLTPAQVVAIASNGGGKQALETVQRLLPVLCQAHGLTPEQVVAIA
SNGGGKQALETVQRLLPVLCQAHGLTPEQVVAIASNGGGKQALETVQRLLPVLCQAH
GLTPEQVVAIASNNGGKQALETVQRLLPVLCQAHGLTPEQVVAIASNIGGRPALESI
VAQLSRPDPALAALTNDHLVALACLGGRPALDAVKKGLG*GS*PTPYPNYANAGHVEGQ
SALFMRDNGISEGLVFHNNPEGTCGFCVNMTETLLPENAKMTVVPPEGAIPVKRGAT
GETKVFTGNSNSPKSPTKGGC*SGGS*TNLSDIIEKETGKQLVIQESILMLPEEVEEVI
GNKPESDILVHTAYDESTDENVMLLTSDAPEYKPWALVIQDSNGENKIKML

H1-G1397C

MLGFVGRVAAAPASGALRRLTPSASLPPAQLLLRAAPTAVHPVRDYAAQTSESGGGG
SPG*AAA*DYKDDDDK*GS*VDLRTLGYSQQQQEKIKPKVRSTVAQHHEALVGHGFTHAHI
VALSQHPAALGTVAVKYQDMIAALPEATHEAIVGVGKQWSGARALEALLTVAGELRG
PPLQLDTGQLLKIAKRGGVTAVEAVHAWRNALTGAPLNLTPEQVVAIASNIGGKQAL
ETVQRLLPVLCQAHGLTPDQVVAIASNIGGKQALETVQRLLPVLCQAHGLTPDQVVA
IASNIGGKQALETVQRLLPVLCQAHGLTPDQVVAIASNIGGKQALETVQRLLPVLCQ
AHGLTPDQVVAIASNNGGKQALETVQRLLPVLCQAHGLTPEQVVAIASNIGGKQALE
TVQRLLPVLCQAHGLTPDQVVAIASNNGGKQALETVQRLLPVLCQAHGLTPEQVVAI
ASNGGGKQALETVQRLLPVLCQAHGLTPEQVVAIASNGGGKQALETVQRLLPVLCQA
HGLTPEQVVAIASNIGGKQALETVQRLLPVLCQAHGLTPDQVVAIASHDGGKQALET
VQRLLPVLCQAHGLTPAQVVAIASNGGGKQALETVQRLLPVLCQAHGLTPEQVVAIA
SNGGGKQALETVQRLLPVLCQAHGLTPEQVVAIASNGGGKQALETVQRLLPVLCQAH
GLTPEQVVAIASNNGGKQALETVQRLLPVLCQAHGLTPEQVVAIASNIGGRPALESI
VAQLSRPDPALAALTNDHLVALACLGGRPALDAVKKGLG*GS*AIPVKRGATGETKVFT
GNSNSPKSPTKGGC*SGGS*TNLSDIIEKETGKQLVIQESILMLPEEVEEVIGNKPESD
ILVHTAYDESTDENVMLLTSDAPEYKPWALVIQDSNGENKIKML

H1-G1333N

MLGFVGRVAAAPASGALRRLTPSASLPPAQLLLRAAPTAVHPVRDYAAQTSESGGGG
SPG*AAA*DYKDDDDK*GS*VDLRTLGYSQQQQEKIKPKVRSTVAQHHEALVGHGFTHAHI
VALSQHPAALGTVAVKYQDMIAALPEATHEAIVGVGKQWSGARALEALLTVAGELRG
PPLQLDTGQLLKIAKRGGVTAVEAVHAWRNALTGAPLNLTPEQVVAIASNIGGKQAL
ETVQRLLPVLCQAHGLTPDQVVAIASNIGGKQALETVQRLLPVLCQAHGLTPDQVVA
IASNIGGKQALETVQRLLPVLCQAHGLTPDQVVAIASNIGGKQALETVQRLLPVLCQ
AHGLTPDQVVAIASNNGGKQALETVQRLLPVLCQAHGLTPEQVVAIASNIGGKQALE
TVQRLLPVLCQAHGLTPDQVVAIASNNGGKQALETVQRLLPVLCQAHGLTPEQVVAI
ASNGGGKQALETVQRLLPVLCQAHGLTPEQVVAIASNGGGKQALETVQRLLPVLCQA
HGLTPEQVVAIASNIGGKQALETVQRLLPVLCQAHGLTPDQVVAIASHDGGKQALET
VQRLLPVLCQAHGLTPAQVVAIASNGGGKQALETVQRLLPVLCQAHGLTPEQVVAIA
SNGGGKQALETVQRLLPVLCQAHGLTPEQVVAIASNGGGKQALETVQRLLPVLCQAH
GLTPEQVVAIASNNGGKQALETVQRLLPVLCQAHGLTPEQVVAIASNIGGRPALESI

VAQLSRPDPALAALTNDHLVALACLGGRPALDAVKKGLG*GS*GSYALGPYQISAPQLP
AYNGQTVGTFYYVNDAGGLESKVFSSGG*SGGS*TNLSDIIEKETGKQLVIQESILMLP
EEVEEVIGNKPESDILVHTAYDESTDENVMLLTSDAPEYKPWALVIQDSNGENKIKM
L

H1-G1397N

MLGFVGRVAAAPASGALRRLTPSASLPPAQLLLRAAPTAVHPVRDYAAQTSESGGGG
SPG*AAA*DYKDDDDK*GS*VDLRTLGYSQQQQEKIKPKVRSTVAQHHEALVGHGFTHAHI
VALSQHPAALGTVAVKYQDMIAALPEATHEAIVGVGKQWSGARALEALLTVAGELRG
PPLQLDTGQLLKIAKRGGVTAVEAVHAWRNALTGAPLNLTPEQVVAIASNIGGKQAL
ETVQRLLPVLCQAHGLTPDQVVAIASNIGGKQALETVQRLLPVLCQAHGLTPDQVVA
IASNIGGKQALETVQRLLPVLCQAHGLTPDQVVAIASNIGGKQALETVQRLLPVLCQ
AHGLTPDQVVAIASNNGGKQALETVQRLLPVLCQAHGLTPEQVVAIASNIGGKQALE
TVQRLLPVLCQAHGLTPDQVVAIASNNGGKQALETVQRLLPVLCQAHGLTPEQVVAI
ASNGGGKQALETVQRLLPVLCQAHGLTPEQVVAIASNGGGKQALETVQRLLPVLCQA
HGLTPEQVVAIASNIGGKQALETVQRLLPVLCQAHGLTPDQVVAIASHDGGKQALET
VQRLLPVLCQAHGLTPAQVVAIASNGGGKQALETVQRLLPVLCQAHGLTPEQVVAIA
SNGGGKQALETVQRLLPVLCQAHGLTPEQVVAIASNGGGKQALETVQRLLPVLCQAH
GLTPEQVVAIASNNGGKQALETVQRLLPVLCQAHGLTPEQVVAIASNIGGRPALESI
VAQLSRPDPALAALTNDHLVALACLGGRPALDAVKKGLG*GS*GSYALGPYQISAPQLP
AYNGQTVGTFYYVNDAGGLESKVFSSGGPTPYPNYANAGHVEGQSALFMRDNGISEG
LVFHNNPEGTCGFCVNMTETLLPENAKMTVVPPEG*SGGS*TNLSDIIEKETGKQLVIQ
ESILMLPEEVEEVIGNKPESDILVHTAYDESTDENVMLLTSDAPEYKPWALVIQDSN
GENKIKML

H2-RVDs

LTPEQVVAIASNIGGKQALETVQRLLPVLCQAHGLTPDQVVAIASNIGGKQALETVQ
RLLPVLCQAHGLTPDQVVAIASNIGGKQALETVQRLLPVLCQAHGLTPDQVVAIASN
IGGKQALETVQRLLPVLCQAHGLTPDQVVAIASNNGGKQALETVQRLLPVLCQAHGL
TPEQVVAIASNIGGKQALETVQRLLPVLCQAHGLTPDQVVAIASNNGGKQALETVQR
LLPVLCQAHGLTPEQVVAIASNGGGKQALETVQRLLPVLCQAHGLTPEQVVAIASNG
GGKQALETVQRLLPVLCQAHGLTPEQVVAIASNIGGKQALETVQRLLPVLCQAHGLT
PDQVVAIASHDGGKQALETVQRLLPVLCQAHGLTPAQVVAIASNGGGKQALETVQRL
LPVLCQAHGLTPEQVVAIASNGGGKQALETVQRLLPVLCQAHGLTPEQVVAIASNGG
GKQALETVQRLLPVLCQAHGLTPEQVVAIASNNGGKQALETVQRLLPVLCQAHGLTP
EQVVAIASNIGGKQALETVQRLLPVLCQAHGLTPDQVVAIASNGGGRPALE

L1-RVDs

LTPEQVVAIASNIGGKQALETVQRLLPVLCQAHGLTPDQVVAIASNIGGKQALETVQ
RLLPVLCQAHGLTPDQVVAIASNNGGKQALETVQRLLPVLCQAHGLTPEQVVAIASN
NGGKQALETVQRLLPVLCQAHGLTPEQVVAIASNNGGKQALETVQRLLPVLCQAHGL
TPEQVVAIASNNGGKQALETVQRLLPVLCQAHGLTPEQVVAIASNNGGKQALETVQR
LLPVLCQAHGLTPEQVVAIASNGGGKQALETVQRLLPVLCQAHGLTPEQVVAIASNG
GGKQALETVQRLLPVLCQAHGLTPEQVVAIASNGGGKQALETVQRLLPVLCQAHGLT

PEQVVAIASNIGGKQALETVQRLLPVLCQAHGLTPDQVVAIASNIGGKQALETVQRL
LPVLCQAHGLTPDQVVAIASNNGGKQALETVQRLLPVLCQAHGLTPEQVVAIASHDG
GKQALETVQRLLPVLCQAHGLTPAQVVAIASNGGGRPALE

L2-RVDs

LTPEQVVAIASNIGGKQALETVQRLLPVLCQAHGLTPDQVVAIASNIGGKQALETVQ
RLLPVLCQAHGLTPDQVVAIASNNGGKQALETVQRLLPVLCQAHGLTPEQVVAIASN
NGGKQALETVQRLLPVLCQAHGLTPEQVVAIASNNGGKQALETVQRLLPVLCQAHGL
TPEQVVAIASNNGGKQALETVQRLLPVLCQAHGLTPEQVVAIASNNGGKQALETVQR
LLPVLCQAHGLTPEQVVAIASNGGGKQALETVQRLLPVLCQAHGLTPEQVVAIASNG
GGKQALETVQRLLPVLCQAHGLTPEQVVAIASNGGGKQALETVQRLLPVLCQAHGLT
PEQVVAIASNIGGKQALETVQRLLPVLCQAHGLTPDQVVAIASNIGGKQALETVQRL
LPVLCQAHGLTPDQVVAIASNNGGKQALETVQRLLPVLCQAHGLTPEQVVAIASHDG
GKQALETVQRLLPVLCQAHGLTPAQVVAIASNGGGKQALETVQRLLPVLCQAHGLTP
EQVVAIASHDGGKQALETVQRLLPVLCQAHGLTPAQVVAIASHDGGRPALE
