# Supplementary material for: Correction of a homoplasmic mitochondrial tRNA mutation in patient-derived iPSCs via a mitochondrial base editor
Source: Commun Biol. 2023 Nov 3;6:1116. doi: 10.1038/s42003-023-05500-y (PMC10624837; doi:10.1038/s42003-023-05500-y)
Supplement: Supplementary file 3 — Description of Additional Supplementary Data [file 42003_2023_5500_MOESM3_ESM.docx]

**Description of Additional Supplementary Files**

**File name:** Supplementary Data 1

**Description:** List of off-targets in each edited clone.

**File name:** Supplementary Data 2

**Description:** List of C∙G-to-T∙A SNVs identified from nuclear genome of edited clones and untreated iPSCs.

**File name:** Supplementary Data 3

**Description:** The source data behind the graphs in the paper
