# Supplementary material for: Correction of a homoplasmic mitochondrial tRNA mutation in patient-derived iPSCs via a mitochondrial base editor
Source: Commun Biol. 2023 Nov 3;6:1116. doi: 10.1038/s42003-023-05500-y (PMC10624837; doi:10.1038/s42003-023-05500-y)
Supplement: Supplementary file 7 — Reporting Summary [file 42003_2023_5500_MOESM7_ESM.pdf]

nature portfolio

Corresponding author(s): Zhengfeng Xu, Jun Zhang, Shiwei Yang, Dong Liang, Bin Shen

Last updated by author(s): Oct 8, 2023

Reporting Summary

Nature Portfolio wishes to improve the reproducibility of the work that we publish. This form provides structure for consistency and transparency in reporting. For further information on Nature Portfolio policies, see our Editorial Policies and the Editorial Policy Checklist.

Statistics

For all statistical analyses, confirm that the following items are present in the figure legend, table legend, main text, or Methods section.

n/a	Confirmed	
☐	☒	The exact sample size (*n*) for each experimental group/condition, given as a discrete number and unit of measurement
☐	☒	A statement on whether measurements were taken from distinct samples or whether the same sample was measured repeatedly
☐	☒	The statistical test(s) used AND whether they are one- or two-sided<br>*Only common tests should be described solely by name; describe more complex techniques in the Methods section.*
☒	☐	A description of all covariates tested
☒	☐	A description of any assumptions or corrections, such as tests of normality and adjustment for multiple comparisons
☐	☒	A full description of the statistical parameters including central tendency (e.g. means) or other basic estimates (e.g. regression coefficient) AND variation (e.g. standard deviation) or associated estimates of uncertainty (e.g. confidence intervals)
☒	☐	For null hypothesis testing, the test statistic (e.g. *F*, *t*, *r*) with confidence intervals, effect sizes, degrees of freedom and *P* value noted<br>*Give P values as exact values whenever suitable.*
☒	☐	For Bayesian analysis, information on the choice of priors and Markov chain Monte Carlo settings
☒	☐	For hierarchical and complex designs, identification of the appropriate level for tests and full reporting of outcomes
☒	☐	Estimates of effect sizes (e.g. Cohen's *d*, Pearson's *r*), indicating how they were calculated

Our web collection on statistics for biologists contains articles on many of the points above.

Software and code

Policy information about availability of computer code

Data collection	Not applicable.
Data analysis	1. For tRNA-seq data analysis, Reads were quality control (QC) by FastQC (v0.11.5) and adapters were trimmed by cutadapt (v1.15). A set of 632 tRNA-reference genes (listed in gtRNAdb) was used for reference and CCA nucleotide sequence was added to the tail if needed. Reads mapping was performed by using Bowtie2 (v2.3.4.1) and alignments with high quality were kept for further analysis. Differential analysis was performed using R package DESeq2 (v1.32.0) and ggplot2 (v2.0.0).<br>2. PRM data were processed with Skyline Daily software.<br>3. For whole genome sequencing analysis, the trimmed reads were mapped to the human reference genome (GRCh38/hg38) by BWA (v0.7.12). Picard-tools (v2.3.0) was used to reorder, sort, add read groups and mark duplicates of the aligned BAM files. Then, Strelka (v2.9.10), Lofreq (v2.1.2) and Mutect2 (v3.8.1) were used to identify the genome-wide de novo variants with high confidence.<br>4. All experiments were repeated at least three times. Statistical analyses were performed with Student's t test or One-way ANOVA using SPSS version 23.0 statistical software (IBM).

For manuscripts utilizing custom algorithms or software that are central to the research but not yet described in published literature, software must be made available to editors and reviewers. We strongly encourage code deposition in a community repository (e.g. GitHub). See the Nature Portfolio guidelines for submitting code & software for further information.

Data

Policy information about availability of data

All manuscripts must include a data availability statement. This statement should provide the following information, where applicable:

- Accession codes, unique identifiers, or web links for publicly available datasets
- A description of any restrictions on data availability
- For clinical datasets or third party data, please ensure that the statement adheres to our policy

The high-throughput sequencing data have been deposited to the NCBI Sequence Read Archive (SRA) database under the accession ID PRJNA921944.

Research involving human participants, their data, or biological material

Policy information about studies with human participants or human data. See also policy information about sex, gender (identity/presentation), and sexual orientation and race, ethnicity and racism.

Reporting on sex and gender	Not applicable.
Reporting on race, ethnicity, or other socially relevant groupings	Not applicable.
Population characteristics	Not applicable.
Recruitment	A one-year and three-month-old boy was admitted to the Children's Hospital of Nanjing Medical University due to recurrent convulsions and lethargy. No pathogenic or likely-pathogenic variants known to associated with this disease were identified in the patient from whole exome sequencing (data not shown), while mitochondrial DNA sequencing identified that the boy carried a homoplasmic m.A4300G mutation in mitochondrial tRNAIle gene.
Ethics oversight	This study was ethically approved by the Medical Ethics Committee of Nanjing Maternal and Child Health Care Hospital (2021KY-131), and informed consents were obtained from the patient's legal guardian as well as the healthy donors, in accordance with the Declaration of Helsinki.

Note that full information on the approval of the study protocol must also be provided in the manuscript.

Field-specific reporting

Please select the one below that is the best fit for your research. If you are not sure, read the appropriate sections before making your selection.

☒ Life sciences ☐ Behavioural & social sciences ☐ Ecological, evolutionary & environmental sciences

For a reference copy of the document with all sections, see nature.com/documents/nr-reporting-summary-flat.pdf

Life sciences study design

All studies must disclose on these points even when the disclosure is negative.

Sample size	No sample-size calculation was performed. All cell samples were evaluated in at least two independent replicate experiments to ensure the reproducibility and the sample size numbers were listed in the corresponding figure legends.
Data exclusions	No data were excluded.
Replication	The experiments were repeated to confirm reproducibility.
Randomization	Cells were randomly assigned into culture plate for transfection.
Blinding	Blinding was not relevant to our study.

Reporting for specific materials, systems and methods

We require information from authors about some types of materials, experimental systems and methods used in many studies. Here, indicate whether each material, system or method listed is relevant to your study. If you are not sure if a list item applies to your research, read the appropriate section before selecting a response.

Materials & experimental systems

n/a	Involved in the study
☐	☒ Antibodies
☐	☒ Eukaryotic cell lines
☒	☐ Palaeontology and archaeology
☒	☐ Animals and other organisms
☒	☐ Clinical data
☒	☐ Dual use research of concern
☒	☐ Plants

Methods

n/a	Involved in the study
☒	☐ ChIP-seq
☐	☒ Flow cytometry
☒	☐ MRI-based neuroimaging

Antibodies

Antibodies used

anti-COX1 (A17889, ABclonal; 1:1000), anti-COX2 (55070-1-AP, Proteintech; 1:1000), anti-COX3 (A9939, ABclonal; 1:1000) and anti-α-Tubulin (AF0001, Beyotime Biotechnology; 1:1000), Horseradish peroxidase (HRP)-linked second antibody (BL001A, Biosharp; 1:5000).

Validation

1. Anti-COX1 (A17889) was verified in human, mouse and rat for western blot.
2. Anti-COX2 (55070-1-AP) were verified in human, mouse, rat, hamster and zebrafish for western blot.
3. Anti-COX3 (A9939) were verified in mouse and rat for western blot.
4. Anti-α-Tubulin (AF0001) were verified in human, mouse and rat for western blot.

Eukaryotic cell lines

Policy information about cell lines and Sex and Gender in Research

Cell line source(s)

HEK293FT cells were bought from Thermofisher (R70007). The iPSC clones used in this study were derived from patient PBMC from both a HCM patient with homoplasmic m.A4300G mutation and a healthy donor.

Authentication

No specific authentication was performed.

Mycoplasma contamination

All cell lines were detected without mycoplasma contamination by PCR test.

Commonly misidentified lines
(See ICLAC register)

No commonly misidentified cell lines were used.

Flow Cytometry

Plots

Confirm that:

☒ The axis labels state the marker and fluorochrome used (e.g. CD4-FITC).

☒ The axis scales are clearly visible. Include numbers along axes only for bottom left plot of group (a 'group' is an analysis of identical markers).

☒ All plots are contour plots with outliers or pseudocolor plots.

☒ A numerical value for number of cells or percentage (with statistics) is provided.

Methodology

Sample preparation

The iPSCs were trypsinized, resuspended in culture medium and sorted via flow cytometry.

Instrument

BD FACSAria™ Fusion SORP sorter

Software

FACS DIVA 8.0

Cell population abundance

EGFP and mCherry double positive rates = double positive cell number / total cell number.

Gating strategy

The cells were first gated based on FSC/SSC and FSC-A/FSC-H to select for live single cells. Untreated cells were employed as negative control for gating EGFP and mCherry signal.

☒ Tick this box to confirm that a figure exemplifying the gating strategy is provided in the Supplementary Information.
